# Human HLA-A*02:01/CHM1^+^ allo-restricted T cell receptor transgenic CD8^+^ T Cells specifically inhibit Ewing sarcoma growth *in vitro* and *in vivo*

**DOI:** 10.18632/oncotarget.9218

**Published:** 2016-05-07

**Authors:** Franziska Blaeschke, Uwe Thiel, Andreas Kirschner, Melanie Thiede, Rebeca Alba Rubio, David Schirmer, Thomas Kirchner, Gunther H.S. Richter, Sabine Mall, Richard Klar, Stanley Riddell, Dirk H. Busch, Angela Krackhardt, Thomas G.P. Grunewald, Stefan Burdach

**Affiliations:** ^1^ Laboratory for Functional Genomics and Transplantation Biology, Department of Pediatrics and Children's Cancer Research Center, Klinikum rechts der Isar, Technische Universitaet Muenchen, Munich, Germany; ^2^ Laboratory for Pediatric Sarcoma Biology, Institute of Pathology of the LMU Munich, Munich, Germany; ^3^ German Cancer Consortium (DKTK), Heidelberg, Germany; ^4^ German Cancer Research Center, Heidelberg, Germany; ^5^ Medizinische Klinik III, Klinikum rechts der Isar, Technische Universität München, Munich, Germany; ^6^ Department of Medicine, University of Washington, Seattle, WA, USA; ^7^ Institute for Medical Microbiology, Immunology and Hygiene, Technische Universitaet Muenchen, München, Germany; ^8^ Focus Group “Clinical Cell Processing and Purification”, Institute for Advanced Study, Technische Universität München, Munich, Germany; ^9^ Munich Comprehensive Cancer Center (CCC), Klinikum rechts der Isar, Technische Universität München, Munich, Germany; ^10^ Present address: Laboratory for Immunotherapy, Dr. von Hauner Children's Hospital, LMU München, Munich, Germany

**Keywords:** Ewing sarcoma, immunotherapy, T cell receptor transgenic T cells, adoptive transfer, allogeneic stem cell transplantation

## Abstract

The endochondral bone protein Chondromodulin-I (CHM1) provides oncogene addiction in Ewing sarcoma (ES). We pre-clinically tested the targetability of CHM1 by TCR transgenic, allo-restricted, peptide specific T cells to treat ES. We previously generated allo-restricted wildtype CD8^+^ T cells directed against the ES specific antigen CHM1^319^ causing specific responses against ES. However, utilization of these cells in current therapy protocols is hampered due to high complexity in production, relatively low cell numbers, and rapid T cell exhaustion.

In order to provide off-the-shelf products in the future, we successfully generated HLA-A*02:01-restricted T cell receptor (TCR) transgenic T cells directed against CHM1^319^ by retroviral transduction.

After short-term expansion a 100% purified CHM1^319^-TCR-transgenic T cell population expressed a CD62L^+^/CD45RO and CD62L^+^/CD45RA^+^ phenotype. These cells displayed specific *in vitro* IFNg and granzyme B release in co-culture with HLA-A*02:01^+^ ES cell lines expressing CHM1. When co-injected with ES cells in Rag2^−/−^ɣc^−/−^ mice, CHM1-specific TCR-transgenic T cells significantly inhibited the formation of lung and liver metastases in contrast to control mice. Lungs and livers of representative mice displayed CD8^+^ T cell infiltration in the presence (control group treated with unspecific T cells) and in the absence (study group) of metastatic disease, respectively. Furthermore, mice receiving unspecific T cells showed signs of graft-versus-host-disease in contrast to all mice, receiving CHM1^319^-TCR-transgenic T cells.

CHM1^319^ specific TCR-transgenic T cells were successfully generated causing anti-ES responses *in vitro* and *in vivo*. In the future, CHM1^319^-TCR-transgenic T cells may control minimal residual disease rendering donor lymphocyte infusions more efficacious and less toxic.

## INTRODUCTION

Oncogene addiction provides ideal targets for immunotherapy. We have described the BRICHOS chaperon domain containing endochondral bone protein Chondromodulin-I (CHM1) being directly up-regulated by the Ewing sarcoma (ES) causing the fusion oncogene product EWS-FLI1. CHM1 maintains an undifferentiated, invasive phenotype and is required for pulmonary metastatic spread of ES [1]. The generation of tumor specific TCR transgenic donor CD8^+^ T cells for adoptive transfer is a promising immunotherapeutic tool to control cancer [2]. Moreover, allorestricted T cell receptor (TCR) responses represent a powerful mechanism in evolution [3]. Given the unknown accessibility of CHM1 by drugs or chimeric antigen receptor transgenic T cells (CARs), we tested the targetability of CHM1 by allo-restricted, peptide specific T cells.

We have already identified the peptide CHM1^319^ as a specific candidate antigen for cancer immunotherapy in an HLA-A*02:01 restricted ES setting [4]. CHM1^319^-specific HLA-A*02:01-restricted T cell clones were identified and expanded *in vitro* and demonstrated good peptide-specificity and tumor control in Rag2^−/−^ɣc^−/−^ mice [4]. Utilization of these cells in current therapy protocols, however, is impaired due to high production complexity, relatively low cell numbers, and rapid T cell exhaustion. In order to overcome these obstacles and to facilitate off-the-shelf ES specific T cells in the future, we generated HLA-A*02:01-restricted TCR transgenic T cells directed against the ES specific antigen CHM1^319^ by retroviral transduction.

Ewing sarcoma (ES) is a highly aggressive malignant tumor with small round blue morphology. The most frequent localizations of disease onset are long bones and pelvis. ES may serve as a paradigm for immunotherapy of hitherto fatal cancer metastatic to bone. Five-year overall survival (OS) of patients with bone or bone marrow metastases at diagnosis and/or early relapse ≤ 24 months after diagnosis is low and does not exceed 15% (advanced ES; AES) [5, 6]. Allogeneic stem cell transplantation is an established treatment for leukemia where donor T cells induce a graft-vs-leukemia response that can eradicate residual malignant cells [7], and is being explored as a treatment for a variety of other hematologic and non-hematologic malignancies [8, 9]. Koscielniak et al. [10] and Lucas et al. [11] reported on AES patients who experienced tumor regression upon allogeneic stem cell transplantation. In recent analyses on the role of allogeneic stem cell transplantation in the treatment of AES patients we demonstrated high treatment toxicity due to graft versus host disease (GVHD) but absence of a graft-versus-ES effect in HLA-matched settings [12, 13]. In a further analysis we demonstrated tumor control in several patients with rhabdomyosarcoma who received unspecific donor lymphocyte infusions (DLI) after allogeneic stem cell transplantation [14]. Taken together, these findings indicate that allogeneic stem cell transplantation may not be sufficient to control cancer by itself, but may serve as platform or model for immunotherapeutic approaches.

## RESULTS

### Wildtype T cell clone CHM1-4B4 specifically recognizes HLA-A*02:01/CHM1^+^ ES cell lines versus controls *in vitro*

**Figure 1:**
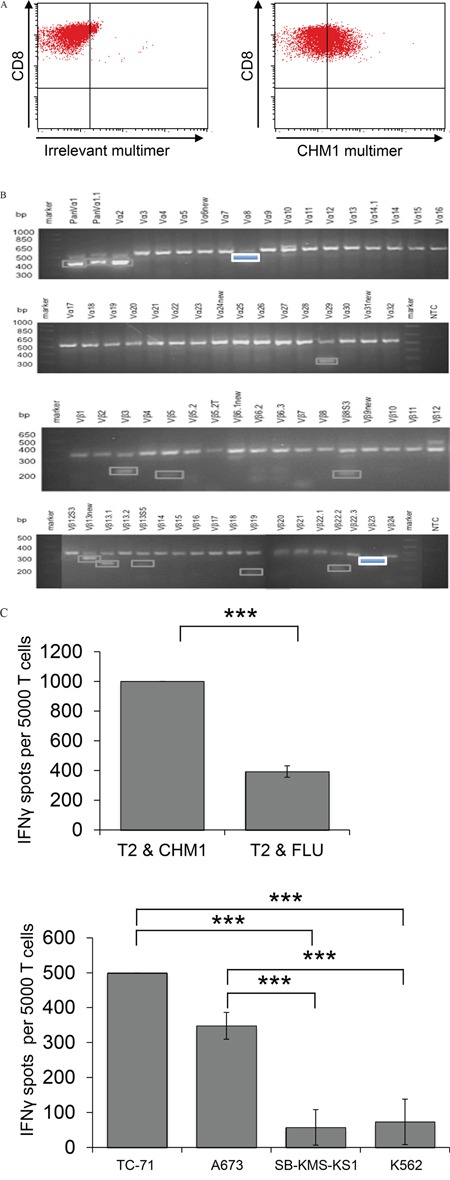
Wild type CHM1-4B4 is clonal and recognizes HLA-A*02:01-restricted CHM1 presentation *in vitro* **A.** Flow cytometric determination of peptide specificity with specific CHM1- HLA-A*02:01-multimers shows CHM1^319^ specificity of wild type CD8+ T cell clone CHM1-4B4. An irrelevant EZH2^666^/HLA-A*02:01-multimer served as control. **B.** CHM1-4B4 was only positive for the variable α-chain TRAV13-1*02, detected by the Vα8 primer and the variable β-chain TRBV13*01, detected by the Vβ23 primer and was thus considered clonal. All other sequenced PCR products were unspecific or showed no open reading-frame (dim bordered boxes). **C.** In IFNγ ELISpot analyses, clone CHM1-4B4 shows peptide specificity and HLA-A*02:01 restriction. A673 and TC71: HLA-A*02:01^+^ ES, SB-KMS-KS1: HLA-A*02:01^−^ ES, K562: MHC^−^ NK cell control. E/T ratio for ELISpot assay: 1:4. Error bars represent standard deviation of triplicate experiments. Asterisks indicate significance levels.

We identified a wildtype CHM1/HLA-A*02:01 restricted CD8^+^ T cell line termed CHM1-4B4 from peripheral blood of a healthy donor. This cell line was tested and stained partially positive with CHM1-HLA-A*02:01-multimer in contrast to irrelevant EZH2^666^-multimer staining (Figure 1A). CHM1-4B4 was only positive for the variable α-chain TRAV13-1*02, detected by the Vα8 primer and the variable β-chain TRBV13*01, detected by the Vβ23 primer and was thus considered clonal. All other sequenced PCR products were unspecific or showed no open-reading-frame (Figure 1B and Supplemental Table 1). CHM1-4B4 T cells recognized T2 cells loaded with CHM1-peptide significantly stronger than T2 cells loaded with an HLA-A*02:01 binding influenza control peptide (FLU, Figure 1C, upper panel) in IFNγ ELISpot assays. Clone CHM1-4B4 secreted IFNγ when presented to CHM1^+^/HLA-A*02:01^+^ cell lines A673 and TC-71 and did not recognize the HLA-A*02:01^−^ cell line SB-KMS-KS1 suggesting peptide specificity and HLA-A*02:01-restriction. The MHC^−^ cell line K562 served as control for natural killer cell (NK) activity and was not recognized.

### Expansion of purified TCR transgenic T cells yield high expansion rates and mediate a partial CD62L/CD45RO^+^ phenotype

**Figure 2:**
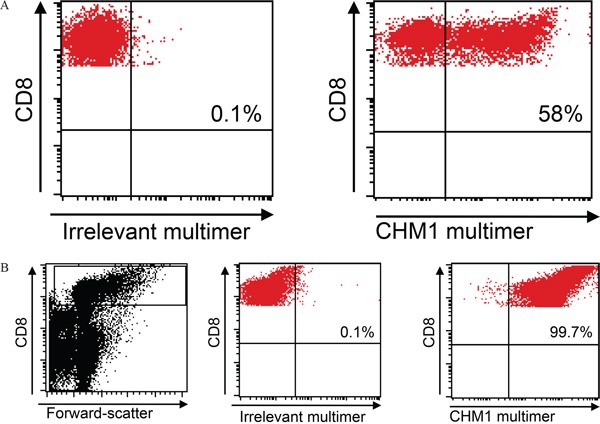
Retroviral transduction and purification **A.** TCR Transduction Flow cytometry reveals a transduction rate of 58% CHM1-TCR^+^ CD8 T cells. **B.** Purification HLA-A*02:01-CHM1^319^-multimer staining reveals nearly 100% CD8^+^ T cell purification after 12 days of expansion. An irrelevant EZH2^666^/HLA-A*02:01 restricted multimer serves as control (live gate).

**Figure 3:**
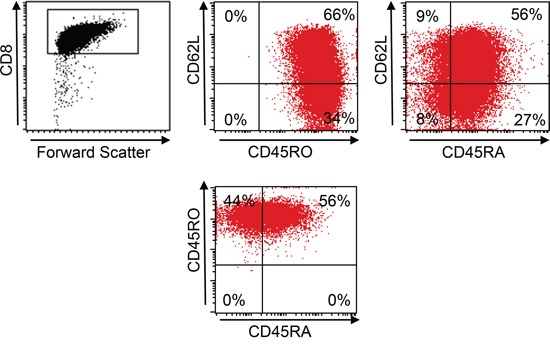
Central memory phenotype of TCR-transgenic T cells At day 12 66% of pMP71_CHM1_mu_opt transduced CD8^+^ T cells from an HLA-A*02:01^−^ healthy donor show a CD62L^+^/CD45RO^+^ central memory (CM) and 56% showed a CD62L^+^/CD45RA^+^ non CM phenotype.

A total of 7 × 10^7^ PBMC from a HLA-A*02:01^−^ healthy donor was used for transduction with the murinized and codon-optimized construct for the CHM1-specific TCR (pMP71_CHM1_mu_opt). Four days after transduction, 58% of all CD8^+^ T cells stained positive for CHM1^319^ multimer in contrast to the irrelevant EZH2^666^ multimer (Figure 2A). After anti-PE-microbead staining and magnetic column isolation, a total of 1 × 10^7^ purified CHM1^319^-TCR-transgenic T cells were expanded over 12 d. At day 12 after transduction, cell counts had reached a total of 1.5 × 10^8^ purified CHM1^319^-TCR-transgenic T cells. All T cells remained nearly a 100% positive for the CHM1 multimer (Figure 2B). At this point, more than 66% of CD8^+^ TCR-transgenic T cells displayed a CD62L^+^/CD45RO^+^ and 56% showed a CD62L^+^/CD45RA^+^ phenotype, indicating a transitional phenotype from naïve to central memory (Figure 3).

### CHM1-specific TCR-transgenic CD8^+^ T cells specifically release IFNγ and granzyme B in co-culture with HLA-A*02:01^+^/CHM1^+^ cell lines *in vitro*

**Figure 4:**
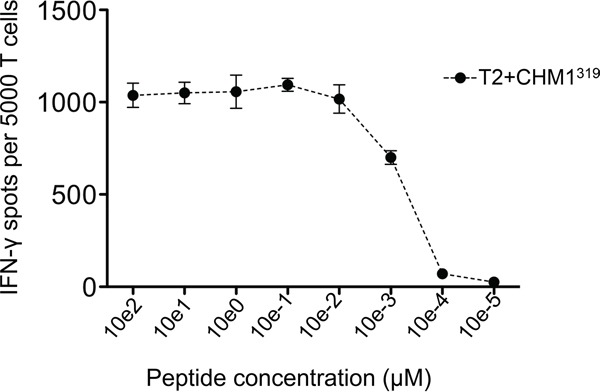
Avidity-testing CHM1^319^-TCR-transgenic T cells recognize CHM1^319^ peptide pulsed on T2 cells in IFNγ ELISpot in a dose dependent manner IFNγ release diminishes at a threshold of <0.01 μM. Data are presented as mean and SEM.

**Figure 5:**
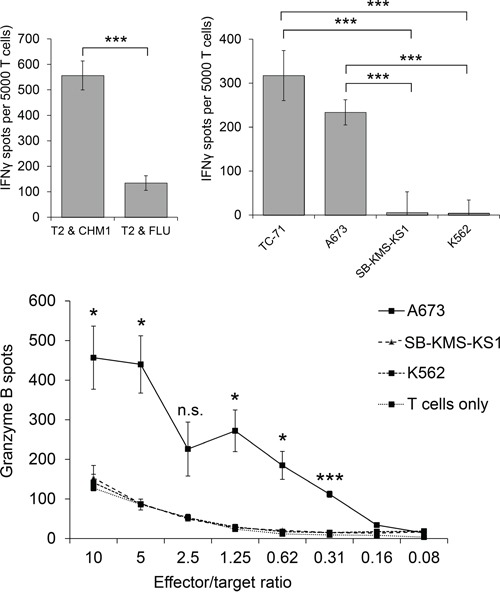
Peptide specificity and HLA-A*02:01-restriction of HLA-A*02:01- CHM1-4B4 TCR-transgenic T cells Specific reactivity against peptide-loaded T2 cells and several tumor cell lines was verified in 5A. IFNγ- and 5B. Granzyme-B ELISpot analyses (A673 and TC71: HLA-A*02:01^+^ ES, SB-KMS-KS1: HLA-A*02:01^−^ ES, K562: MHC^−^ NK cell control,). Effector/target ratio for IFNγ-ELISpots is 1:4. Error bars represent standard deviation of triplicate experiments. Asterisks indicate significance levels; N.s., not significant.

HLA-A*02:01^−^ CD8^+^ T cells transduced with the CHM1^319^-specific construct (pMP71_CHM1_mu_opt) were tested for functionality in ELISpot assays. Processing and transport of the predicted CHM1^319^ nonamer to the surface of target cells was previously demonstrated using HLA-A*02:01 and CHM1 double-transfected Cos7 cells [4]. We determined avidity of CHM1^319^-TCR-transgenic T cells by down-titration of CHM1^319^ peptide onto T2 cells and observed decreasing amounts of IFNγ release at a threshold of <0.01 μM (Figure 4). In accordance with the results obtained for the CHM1-4B4 wildtype clone, CHM1^319^-TCR-transgenic T cells remained specific against CHM1^319^ loaded T2 cells and HLA-A*02:01^+^/CHM1^+^ ES cell lines compared to controls in IFNγ ELISpot assays (Figure 5A) and showed no cross-reactivity against control LCL covering the most common HLA antigens of the HLA-A2 superfamily as described before [17] (Supplemental Figure 2). In granzyme B ELISpot assays CHM1-specific TCR-transgenic T cells specifically released granzyme B upon co-culture with the HLA-A*02:01^+^/CHM1^+^ ES cell line A673 in a dose-dependent manner in contrast to controls (Figure 5B).

### Adoptive transfer of CHM1-specific TCR-transgenic T cells significantly reduce Ewing sarcoma liver metastases in Rag2^−/−^_ɣc_^−/−^ mice versus controls

To investigate *in vivo* efficacy of CHM1-specific TCR-transgenic T cells, their ability to inhibit tumor growth was tested in a preclinical mouse model. Twenty-one days after i.v. co-injection of A673 ES cell lines alone (control group 1, n=5) or in combination with either human PBMC including unspecific T cells (control group 2, initially n=10) or CD8^+^ depleted/CHM1^319^-TCR-transgenic T cells repleted PBMCs (study group, n=9), Rag2^−/−^ɣ_C_^−/−^ mice were sacrificed and analyzed. To this point two out of ten control group 2 mice had died four (mouse #10) and ten (mouse #13) days after A673 ES/PBMC injection, respectively. These mice showed massive stomach bleeding and gastric mucositis as well as mesenteritis in the presence of CD3^+^ and CD8^+^ T cell infiltration in line with the presence of GvHD. Representative data of gastric mucosa of mouse #13 is shown in Supplemental Figure 3. Both mice showed tumor-free lungs and livers and were censured due to early treatment related death.

**Figure 6:**
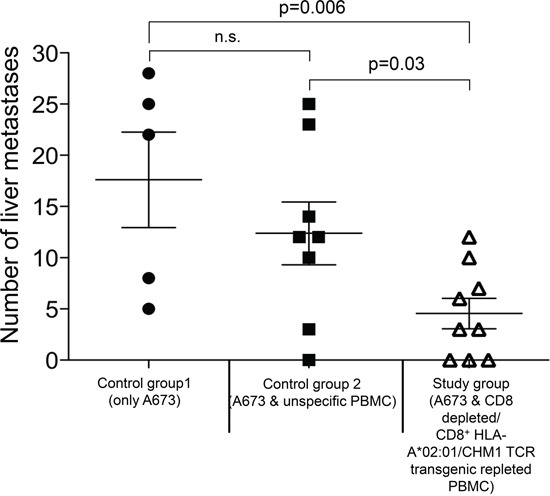
Adoptive Transfer CHM1319-TCR-transgenic CD8+ T cells treated Rag2−/−ɣc−/− mice have significantly lower tumor burden On day 21 after adoptive transfer Rag2^−/−^_ɣc_^−/−^ mice treated with CHM1^319^-TCR-transgenic T cells (study group) show significantly lower numbers of liver metastases on the organ surface in contrast to mice receiving no T cells (control group 1, p=0.006) and mice receiving unspecific T cells (control group 2; p=0.03). There is no statistical difference between both control groups. N.s., not significant.

**Figure 7:**
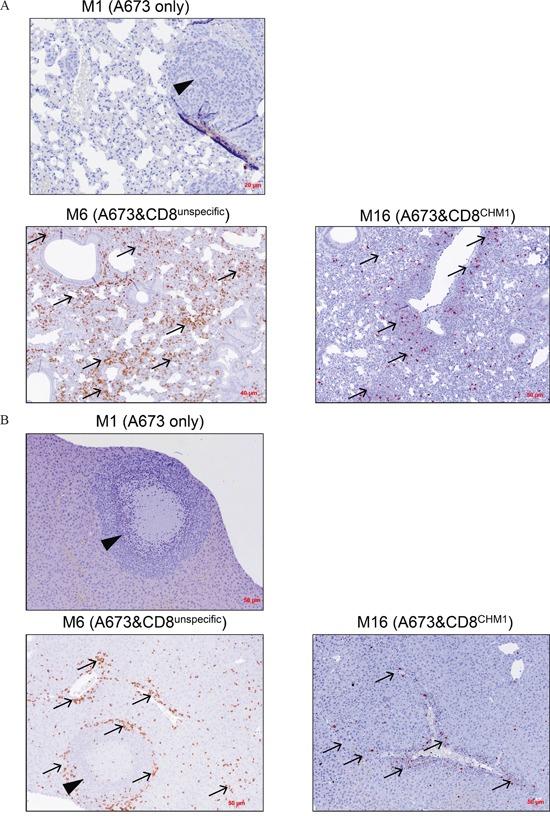
HLA-A*02:01/CHM1+ allo-restricted T Cell Receptor Transgenic CD8+ T Cells cause CD8+ infiltration in liver and lung tissue Immuno-histochemical analysis of exemplary mice from each group reveals tumor growth in **A.** lungs and **B.** liver as well as CD8+ absence in mouse #1 (M1, no T cell treatment). On day 21 after i.v. adoptive transfer, an invasion of CD8^+^ T cells in tumor-free lungs and metastatic liver of mouse #6 (M6, treated with unspecific T cells) as well as in tumor-free lungs and tumor-free liver in mouse #16 (M16, treated with CHM1^319^-TCR-transgenic CD8^+^ T cells, short: CD8^CHM1^) can be observed, respectively. Arrows, CD8^+^ T cells; arrowheads, ES metastasis.

In control group 1 mice, livers (and lungs; data not shown) showed explicit metastatic disease in contrast to control group 2 and study group mice, where only livers were affected. Three mice receiving CHM1^319^-TCR-transgenic T cells and one mouse receiving unspecific T cells were tumor-free at the date of data censure. Study group mice showed significantly lower numbers of liver metastases on the organ surface compared to those of both control groups (P<0.05) (Figure 6). These findings were exemplarily confirmed after calculation of tumor areas in sectioned livers of three representative mice from each group. Only the differences between control group 1 and control group 2 mice versus study group mice were statistically significant (p>0.05; Supplemental Figure 4). Immunohistochemical staining revealed a strong invasion of CD8^+^ T cells in livers (Figure 7A) and lungs (Figure 7B) of mouse #6 and mouse #16 and T cell absence in mouse #1 that had not received any T cell treatment. Interestingly, the CD8^+^ T cell invasion in control group mouse #6 was stronger than in study group mouse #16, a finding that may reflect a stronger but still none-protective immune response of unspecific T cells compared CHM1^319^-TCR-transgenic T cells. Altogether, CD8^+^ T cell infiltration was significantly higher in the proximity of liver tumor lesions in contrast to normal liver parenchyma in 5 out of 7 representative control group 2 and study group mice (study group mouse #16 showed no tumor tissue in the liver; Supplemental Figure 5).

## DISCUSSION

Loss of target antigen is a major mechanism of resistance to immunotherapy of cancer, e.g. loss of CD19 expression during CD19 directed CAR T cells in leukemia treatment [20]. Thus, efficacious T cell immunotherapy needs to address targets obligatory for cancer cell survival. Oncogenes essential for cancer survival constitute ideal targets for immunotherapy. The BRICHOS chaperon domain containing endochondral bone protein CHM1 is being directly up-regulated by the fusion oncogene product EWS-FLI1 causing ES. CHM1 maintains an undifferentiated, invasive phenotype and is required for pulmonary metastatic spread and therefore constitutes such an addictive oncogene [1]. Adoptive immunotherapy with autologous or allogeneic donor T cells against such targets has proven a promising tool to eradicate residual disease in some patients resistant to conventional therapy regimens [2, 7, 21]. In the autologous setting, tumor-infiltrating lymphocytes from tumor samples of melanoma patients showing distinct phenotype patterns induced partial and complete anti-tumor responses probably against antigens derived from immunogenic somatic mutations. Some reports indicate that the lessons learned from immunotherapy of melanoma patients could be potentially translated to other solid tumor entities [9]. However, in the pediatric sarcoma setting, tumor immunogenicity remains yet to be determined [4, 6, 13, 14, 22–25]. The possibility to target tumor cell antigens essential for survival and proliferation, including transcription factors such as CHM1 renders the use of peptide specific T cells is very attractive. We have shown that CHM1 loss is a clear disadvantage for ES. Antigen loss has been observed in CARs studies resulting in relapse [20]. However, reproducible anti-tumor responses in other solid tumors remain scarce, possibly due to lacking immunogenicity of over-expressed target antigens and/or the presence of tumor-mediated immune suppression despite prior lympho-ablative regimen [8, 23, 26, 27]. Furthermore, transfusion of supposedly promising TCR transgenic T cells may reveal severe and fatal off-target effects due to cross-reactivity [28].

In the pediatric setting, allogeneic stem cell transplantation in solid tumors is a matter a debate [7, 14]. Thus additional therapeutic strategies exploiting the power of allo responses are warranted. To date, efficacy of donor lymphocyte infusions could not be unequivocally proven in prospective trials. A further drawback in the administration of unspecific donor lymphocytes is the risk of severe autoimmune toxicity such as life-threatening GvHD [6, 14]. The administration of donor lymphocytes with a TCR directed against tumor-associated antigens constitutes a rationale to circumvent GvHD. However, generation of wild type T cells against ES specific peptides is laborious, expensive, time consuming and cell counts are unstable [4]. Furthermore, at the time of administration T cell function may have exhausted to some degree due to extending division cycles. In a previous work we demonstrated the generation of HLA-A*02:01/CHM1^+^ CD8^+^ T cells specifically lysing ES cells. However, cell numbers were low and T cells carried a differentiated effector phenotype. *In vivo* CD8^+^ T cell activity after i.v. application was weak and transferred T cells could not be tracked possibly due to the lack lymphatic organs and of an adequate cytokine environment [4].

In this work, we identified a new TCR that recognizes the ES specific CHM1^319^ antigen. Processing and transport of the predicted CHM1^319^ nonamer peptide to the surface of target cells using HLA-A*02:01 and CHM1 double-transfected Cos7 cells was shown before by our group [4]. Despite some background IFNγ release upon co-culture with T2 cells loaded with unspecific FLU antigen, we were able to show specific IFNγ and granzyme B release in response to CHM1 expressing ES cell lines. Background reactivity may be explained by the presence of weak allo-recognition that unmask in the artificial T2 setting due to particularly dense peptide/MHC presentation on these cells compared to control tumor cell lines. This assumption is supported by our observation that the flow-cytometric HLA-A2 signal is several fold higher on T2 cells in contrast to HLA-A2 positive ES cell lines that have to be treated with IFNγ to enhance HLA-A*02:01 expression prior to ELIspot analyses [4]. Furthermore, T cell mediated IFNγ release is repeatedly stronger in co-culture with CHM1^319^ loaded T2 cells compared to ES cell lines, again indicating a non-physiological setting. After all, background reactivity did not translate into cross-reactivity as demonstrated in Supplemental Figure 2. Moreover, unspecific FLU recognition was not observed when CHM1^319^-TCR-transgenic T cells were tested in IFNγ and granzyme B Elispot assays. Using microbead-mediated column separation of multimer positive CHM1^319^-TCR-transgenic T cells we could enrich the CHM1^319^-TCR-transgenic T cells up to nearly 100%. Furthermore, we demonstrate that large-scale generation of ES specific CHM1^319^-TCR-transgenic T cells is reproducibly feasible within a short time frame. CHM1^319^-TCR-transgenic T cells displayed a CD62L^+^/CD45RO^+^ and CD62L^+^/CD45RA^+^ phenotype, indicating a transitional phenotype from naïve to central memory. After co-injection with A673 ES cells and CD8 depleted PBMC i.v. in immune-deficient Rag2^−/−^ɣ_C_^−/−^ mice, CHM1^319^-TCR-transgenic T cells were able to significantly inhibit formation of lung and liver metastases compared to controls and to survive for at least 21 days. Furthermore, two mice receiving unspecific T cells showed signs of GVHD in contrast to all study group mice, which tolerated transfer of CHM1^319^-TCR-transgenic T cells well.

CHM1 is an essential oncogene responsible for ES survival. In this work we demonstrate ES susceptibility to treatment with CHM1^319^-TCR-transgenic T cells. The present endeavors constitute a first step to facilitate future off-the-shelf generation of tumor specific T cells targeting CHM1 to eradicate residual disease in AES patients with hitherto poor prognosis. Adequate preparative chemotherapy protocols and *Good Manufacturing Practice* compatible *ex vivo* expansion protocols to induce CM or stem cell memory T cells [29–31] are warranted to translate our approach into the clinical setting.

## MATERIALS AND METHODS

### Cell lines

The HLA-A*02:01^+^ ES cell line TC-71 was obtained from the German Collection of Microorganisms and Cell Cultures (DSMZ, Braunschweig, Germany). A673 (HLA-A*02:01^+^ ES cells) were from ATCC (LGC Standards GmbH, Wesel, Germany). TAP-deficient HLA-A*02:01^+^ T2 cells were obtained from P. Cresswell (Yale University School of Medicine, New Haven, CT, USA). The MHC^−^ erythroid leukemia cell line K562 was a gift from A. Knuth and E. Jäger (Krankenhaus Nordwest, Frankfurt, Germany). HLA-A*02:01^−^ SB-KMS-KS1 cells were previously described by our laboratory [15, 16]. All cell lines were tested for mycoplasma contamination and purity. Lymphoblastoid cell lines (LCL) were generated by EBV transformation of peripheral blood B cells from HLA-A*02:01^−^ healthy donors by use of a mini-EBV plasmid. LCL of German Caucasian HLA-A types were used for cross-reactivity testing and were a kind gift by S. Marsh (Anthony Nolan Research Institute, University College London). Cell lines were cultured as described before [4].

### CHM1 overexpression in Ewing sarcoma

We confirmed relative CHM1 over-expression [4] in primary ES samples compared to healthy tissue samples using the R2: microarray analysis and visualization platform (http://r2.amc.nl) (Supplemental Figure 1).

### Isolation and ELISpot assay of wildtype HLA-A*02:01/CHM1 restricted CD8^+^ T cells

Peptide-specific wildtype T cells were isolated and expanded as previously described by our group [4]. Peripheral blood mononuclear cells (PBMC) were isolated from buffy coat bags of healthy donors (obtained with IRB approval and informed consent from the DRK-Blutspendedienst Baden-Württemberg-Hessen in Ulm, Germany) using Ficoll-Paque density gradient centrifugation according to the supplier's information (GE Healthcare, Uppsala, Sweden). Isolation of CD8^+^ T cells was performed using the CD8^+^ T cell isolation kit according to the manufacturer's instructions (Miltenyi Biotec, Bergisch Gladbach, Germany). *In vitro* priming, multimer-mediated cell sorting and expansion of wildtype T cells was conducted as described before [4]. Expanded HLA-A*02:01/CHM1 restricted CD8^+^ T cells were further characterized in ELISpot assays according to the supplier's information.

### Identification of specific TCR

RNA was extracted from T cells using TriReagent according to the supplier's information (Sigma-Aldrich, St. Louis, Missouri, USA) and transcribed into cDNA with the help of High Capacity Reverse Transcription Kit (Life Technologies). cDNA was used as a template for PCR analysis of TCR chains as previously described [17]. 34 different primers were used for detection of the variable α-chain (Vα) and 35 different primers for detection of the variable β-chain (Vβ) [17]. The corresponding 3′-primers for the variable chain analysis were 3′TCα (α-chain) and 3′CβII (β-chain). Control bands were flanked by the constant primers 3′αST and 5′αST (α-chain) and 3′βST and 5′βST (β-chain). AccuPrime® Taq DNA Polymerase System (Life Technologies) was used for PCR reaction. Positive PCR bands on agarose gels were cut out, extracted (StrataPrep DNA Gel Extraction Kit, Agilent Technologies) and sequenced. Primers used are listed in Supplemental Table 1.

### TCR cloning and retroviral transduction

The murinized and codon-optimized TCR construct (pMP71_CHM1_mu_opt) was generated using the GeneART® Gene Synthesis web tool (Life Technologies): Human constant chains were exchanged for the murine counterpart and codon-optimization of the whole insert was performed as previously described [18]. Inserts were cloned into the retroviral vector pMP71. Healthy donor derived CD8^+^ T cells were transduced as previously described [18].

Briefly, the packaging cell line RD114 was seeded 24 h before transfection into 6 well plates. Transfection was performed using TransIT-293T according to manufactures manual. 9 μf of TransIT were added, vortexed, and incubated at RT for 20 min. 1 μg of TCR plasmid was added and mixed carefully. After 30 min of incubation solution was added dropwise onto the cells and incubated for 48 h at 37°C. Virus containing supernatant was collected, centrifuged at 1500 rpm for 5 min and steril filtered (0,45 μm). PBMCs/T-cells for viral transduction were isolated from Buffy coats and stimulated with 50 ng/ml OKT-3 and 100 U/ml 48h prior to spin infection. The day before transduction non-treated 24-well plates were coated with 400 μl Retronectin in PBS at a concentration of 12,5 μg/ml and stored at 4°C. Wells were blocked with 2% BSA in PBS for 1 h at 37°C and then washed twice with 2.5% HEPES in PBS. Stimulated PBMCs/T-cells were collected and set to a concentration of 1×10^6^ / ml in TCM. 1 ml of each Virus supernatant and T cells were added into coated 6-well plates with additional Protamine-sulfate (c_end_ = 4 μg / ml), HEPES (c_end_ = 0.5%), and IL-2 (c_end_ = 100 U / ml). Plates were centrifuged for 90 min at 820 g in at 32°C preheated centrifuge and stored at 37°C over night. The next day cells were harvested and split 1 : 1. Cells were again placed on coated 6-well plates with fresh virus plus additives and centrifuged at 820 g/90 min/37°C. Medium was replaced after 48 h and transduction efficacy was determined after 72 h via multimer staining. Sequences are listed in Supplemental Table 1.

### Staining, isolation and purification of CHM1^319^-TCR-transgenic T cells

To evaluate the phenotype of TCR-transgenic T cells, flow cytometry staining was performed on a FACS Calibur (BD Biosciences) using specific peptide- HLA-A*02:01-multimers as described before [19]. Cells were stained for a PE labeled CHM1^319^ specific peptide- HLA-A*02:01-multimer and counterstained for CD8-FITC. An irrelevant peptide- HLA-A*02:01-multimer (for analysis of CD8^+^CHM1^319^ specificity) and isotype FITC-, PE- and APC labeled IgG mAb (for all staining other than multimer staining, all events localized in the lower left quadrant; data not shown) served as negative controls. On day 6 after transduction, TCR-transgenic T cells were stained for peptide-HLA-A*02:01-multimer and purified with anti-PE beads according to the supplier's information (Miltenyi Biotec, Bergisch Gladbach, Germany).

### Expansion of TCR-transgenic T cells

After selection via magnetic bead separation, CHM1^319^-TCR-transgenic T cells were cultured in 75 cm^2^ cell culture flasks (TPP, Trasadingen, Switzerland) with 40 ml T cell medium supplemented with IL-2 (3000 IU / ml) and irradiated PBMCs pooled from 3 different healthy donors (3 × 10^8^ / flask) for 6 days. Subsequently T cells were cultured in 25 cm^2^ cell culture flasks (TPP, Trasadingen, Switzerland) with anti-CD3 (30 ng / ml), IL-2 (100 IU / ml) and IL-15 (2 ng / ml; interleukines were added every other day for 12 days), irradiated LCL; 5 × 10^6^ / flask and irradiated PBMCs pooled from 3 different healthy donors (2.5 × 10^7^ / flask) as feeder cells as described before [4].

### Adoptive T-cell transfer in Rag2^−/−^_ɣc_^−/−^ mice

Immunodeficient Rag2^−/−^_γc_^−/−^ mice on a BALB/c background were obtained from the Central Institute for Experimental Animals (Kawasaki, Japan). Mice were bred and maintained in our animal facility under pathogen-free conditions in accordance with the institutional guidelines and approval to local authorities. On day −1 all mice were irradiated with 3,5 Gy. On day 0, each mouse received 2.5 × 10^6^ A673 ES cells intravenously (i.v.). Immediately after A673 injection, mice were either left untreated (control group 1, n = 5) or were treated by i.v. injection of 1 × 10^7^ PBMC containing 2 × 10^6^ unspecific T cells (control group 2, n = 10) or treated with a total of 1 × 10^7^ CD8^+^ T cell depleted and 2 × 10^6^ CHM1^319^-TCR-transgenic T cell repleted PBMC (study group, n = 9), respectively, using the MACS negative isolation kit according to the manufacturer's instructions (Miltenyi Biotec, Bergisch Gladbach, Germany). 1 × 10^7^ IL-15 producing NSO cells were irradiated at 80 Gy and injected intra-peritoneally (i.p.) twice a week to all mice. On day 21 after injection of A673 cells, mice were sacrificed, superficial lung and liver metastases were counted and histo-pathological analyses were performed.

### (Immune-)Histo-pathological analyses

In representative mice liver and lungs sectioned and haematoxylin/eosin (HE) and CD8 stained. Mean calculation of Ewing sarcoma tissue versus healthy parenchyma per individual organ was calculated following serial organ sectioning of representative mice. CD8^+^ T cell infiltration was calculated by counting mean CD8^+^ T cell numbers per 40x magnification field here defined as high power field (HPF) in the proximity of tumor lesions versus healthy parenchyma.

### Statistical analysis

Descriptive statistics was used to determine mean, standard deviation and standard error of the mean (SEM). Differences were analyzed by unpaired two-tailed student's t-test as indicated using Excel (Microsoft) or Prism 5 (GraphPad Software); *p* values < 0.05 were considered statistically significant (*p < 0.05; **p < 0.005; ***p < 0.0005).

## SUPPLEMENTARY FIGURES AND TABLE




