# Supplementary material for: Human HLA-A*02:01/CHM1+ allo-restricted T cell receptor transgenic CD8+ T Cells specifically inhibit Ewing sarcoma growth in vitro and in vivo
Source: Oncotarget. 2016 May 7;7(28):43267–80. doi: 10.18632/oncotarget.9218 (PMC5190022; doi:10.18632/oncotarget.9218)
Supplement: Supplementary file 1 [file oncotarget-07-43267-s001.pdf]

Human HLA-A*02:01/CHM1⁺ allo-restricted T cell receptor transgenic CD8⁺ T Cells specifically inhibit Ewing sarcoma growth *in vitro* and *in vivo*

SUPPLEMENTARY FIGURES AND TABLE

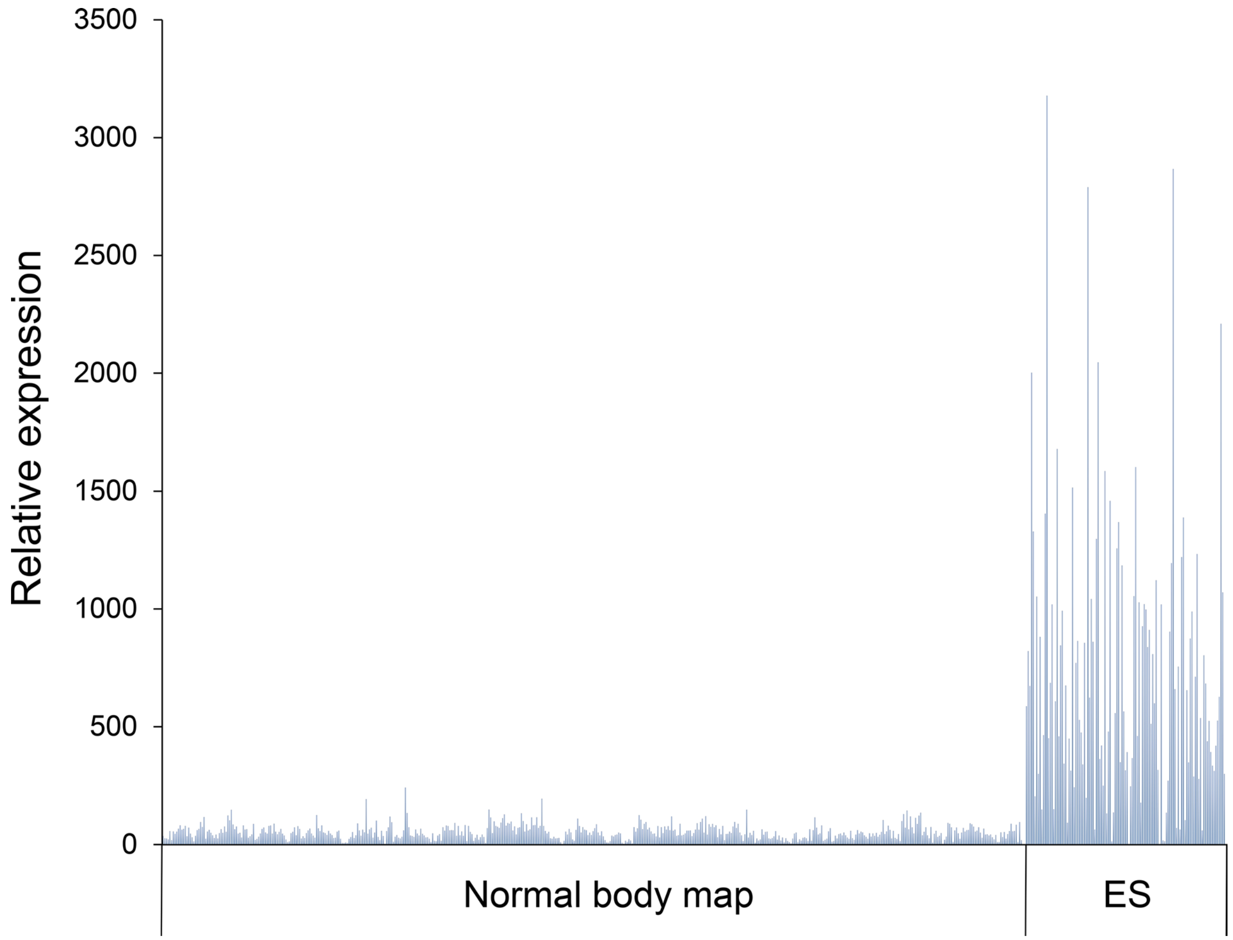

Supplementary Figure S1: CHM1 Expression in ES versus normal body map. Relative gene expression levels of CHM1 in healthy tissue samples (Normal Body Map, left panel; n=504) compared to primary ES samples (ES, right panel; n=117,) [32]. Data were retrieved from the R2: microarray analysis and visualization platform (http://r2.amc.nl).

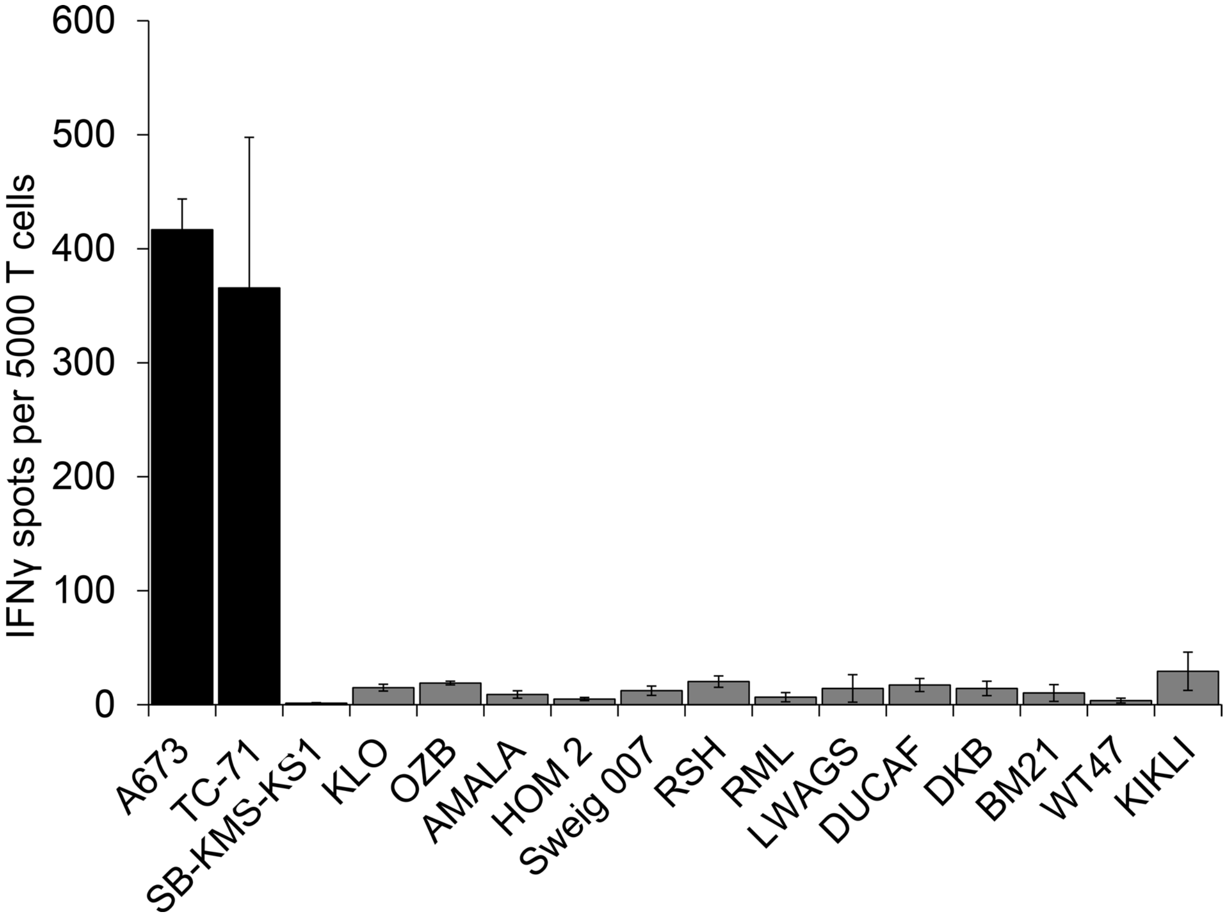

Supplementary Figure S2: No Cross-reactivity of CHM1^{319}-TCR-transgenic CD8^{+} T cells. LCL covering the most common HLA antigens of the HLA-A2 superfamily were used as target cells for CHM1^{319}-TCR-transgenic CD8^{+} T cells at an effector-target ratio of 1:4. Error bars indicate standard deviation of tested triplicates. HLA-A*02:01^{+} ES cell lines A673 and TC-71, and the HLA-A*02:01^{-} ES cell line SB-KMS-KS1 were used as positive and negative controls.

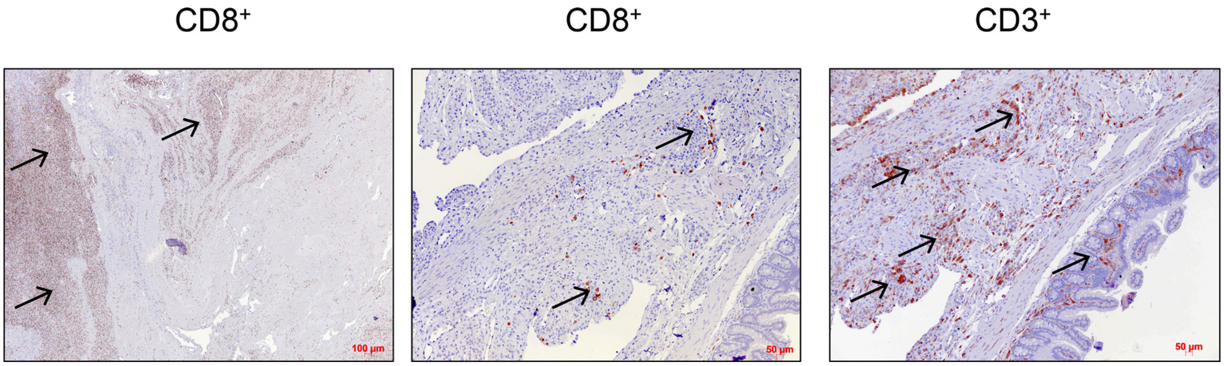

Supplementary Figure S3: Adoptive transfer of unspecific T cells cause gastric GvHD. Immunohistochemical staining reveals swollen gastric walls and mesenteric tissue in the presence of a strong invasion of $CD3^+$ T cells containing a sub-population of $CD8^+$ T cells (arrows) of exemplary mouse #13 (study group) ten days after i.v. adoptive transfer of 1 x 10^7 unspecific PBMC (control group 2) in line with the presence of GvHD.

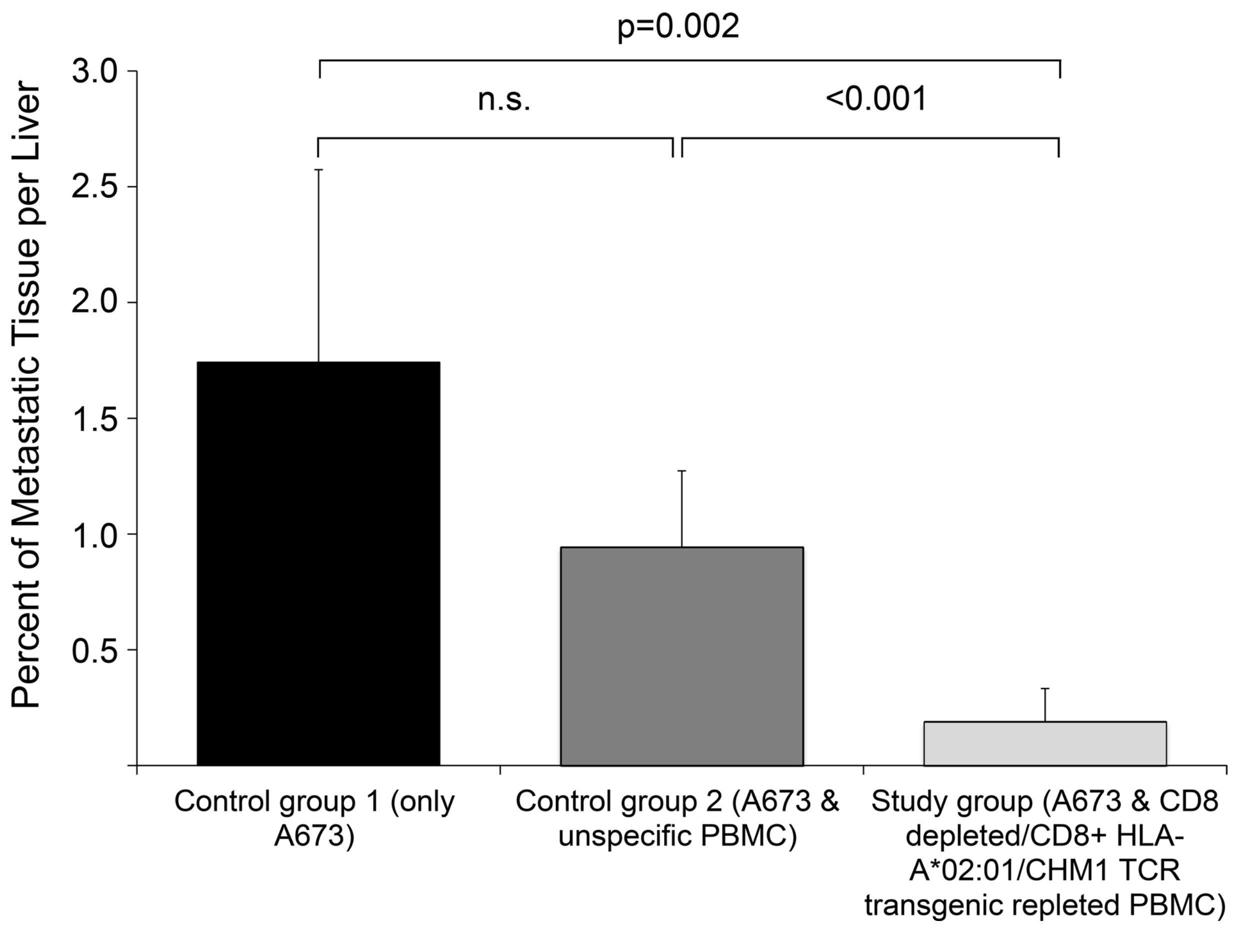

Supplementary Figure S4: Ewing sarcoma burden is proves significantly lower in representative study group mice versus controls. Histologically calculated mean tumor areas in relation to respective organ areas of sectioned livers of three representative mice per group. Only the differences between control group 1 and control group 2 mice versus study group mice were statistically significant ($p < 0.05$).

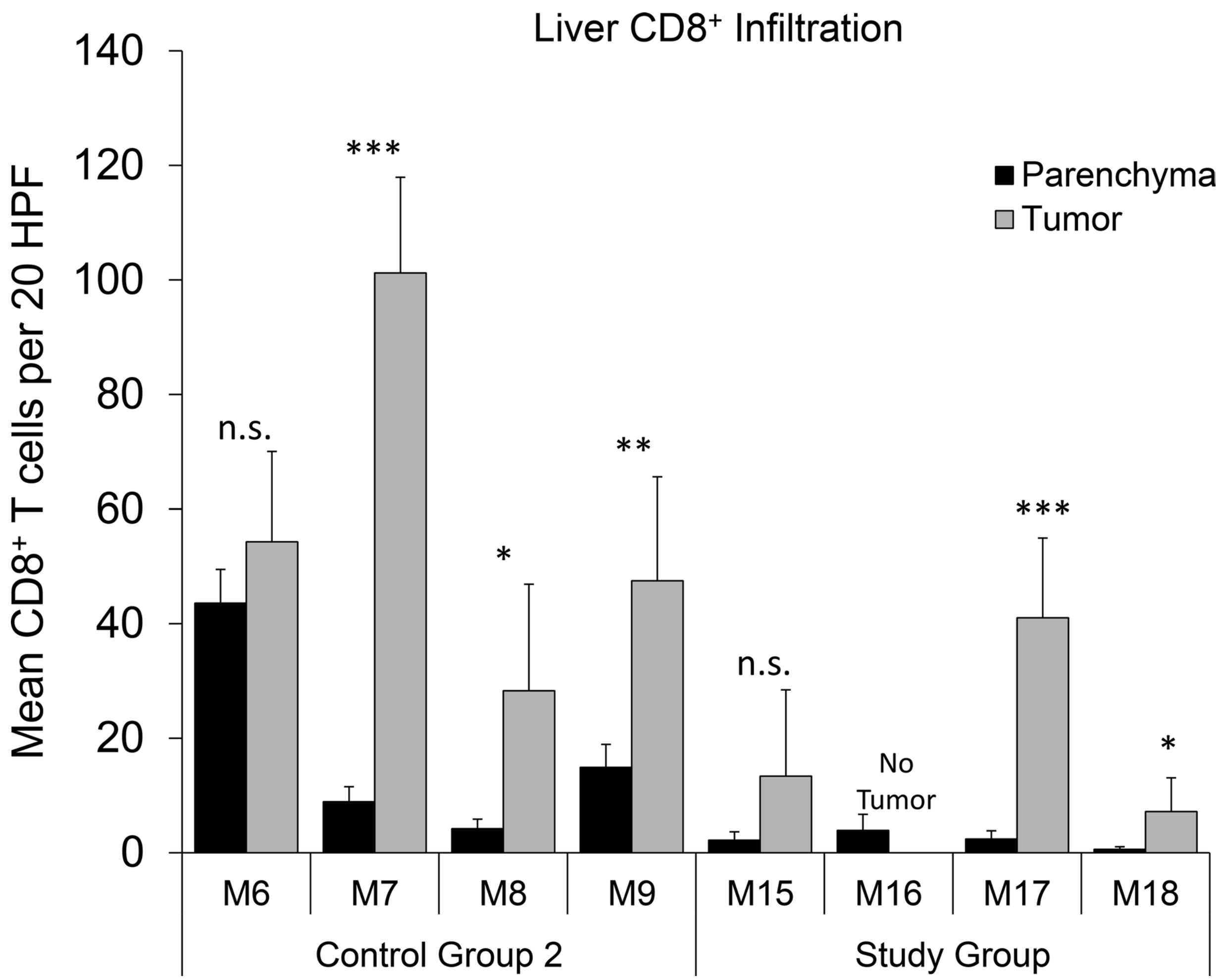

Supplementary Figure S5: CD8$^+$ T cell home to Ewing sarcoma lesions in 5 out of 7 representative mice. Five out of seven representative control group 2 and study group mice show significantly higher CD8$^+$ T cell infiltration in the proximity of tumor lesions (as calculated by CD8$^+$ T cells per 40x magnification defined high power field (HPF) (study group mouse #16 showed no tumor tissue in the liver). *p* values < 0.05 were considered statistically significant (*$p < 0.05$; **$p < 0.005$; ***$p < 0.0005$).

Supplementary Table S1: CHM1 TCR sequences. A. Unmodified sequence of the variable part of the α-chain TRAV13-1*02; B. Unmodified sequence of the variable part of the β -chain TRAV13*01; C. Murinized and optimized sequence of the insert used for construction of pMP71_CHM1_mu_opt.

See Supplementary File1
