# Supplementary material for: Human HLA-A*02:01/CHM1+ allo-restricted T cell receptor transgenic CD8+ T Cells specifically inhibit Ewing sarcoma growth in vitro and in vivo
Source: Oncotarget. 2016 May 7;7(28):43267–80. doi: 10.18632/oncotarget.9218 (PMC5190022; doi:10.18632/oncotarget.9218)
Supplement: Supplementary file 2 [file oncotarget-07-43267-s002.docx]

| A.  Unmodified sequence of the variable part of the α-chain (TRAV13-1*02). | atgacatccattcgagctgtatttatattcctgtggctgcagctggacttggtgaatggagagaatgtggagcagcatccttcaaccctgagtgtccaggagggagacagcgctgttatcaagtgtacttattcagacagtgcctcaaactacttcccttggtataagcaagaacttggaaaaagacctcagcttattatagacattcgttcaaatgtgggcgaaaagaaagaccaacgaattgctgttacattgaacaagacagccaaacatttctccctgcacatcacagagacccaacctgaagactcggctgtctacttctgtgcagcaagcgctggaggaagccaaggaaatctcatctttggaaaaggcactaaactctctgttaaaccaaAT |
| --- | --- |
| B.  Unmodified sequence of the variable part of the β-chain (TRBV13*01). | atgcttagtcctgacctgcctgactctgcctggaacaccaggctcctctgccgtgtcatgctttgtctcctgggagcaggttcagtggctgctggagtcatccagtccccaagacatctgatcaaagaaaagagggaaacagccactctgaaatgctatcctatccctagacacgacactgtctactggtaccagcagggtccaggtcaggacccccagttcctcatttcgttttatgaaaagatgcagagcgataaaggaagcatccctgatcgattctcagctcaacagttcagtgactatcattctgaactgaacatgagctccttggagctgggggactcagccctgtacttctgtgccagcagctttctgggggaaaaaactgaagctttctttggacaaggcaccagactcacagttgtagAG |
| C.  Murinized and optimized sequence containing α- and β-chain (insert “CHM1_mu_opt“). | CACTATAGGGCGAATTGGCGGAAGGCCGTCAAGGCCGCATGCGCGGCCGCCACCATGCTGTCTCCAGATCTGCCTGACAGCGCCTGGAACACCCGGCTGCTGTGCAGAGTGATGCTGTGCCTGCTGGGAGCCGGATCTGTGGCTGCTGGCGTGATCCAGAGCCCCAGACACCTGATCAAAGAGAAGAGAGAGACAGCCACCCTGAAGTGCTACCCCATCCCCAGGCACGACACCGTGTACTGGTATCAGCAGGGCCCAGGCCAGGACCCCCAGTTCCTGATCAGCTTCTACGAGAAGATGCAGAGCGACAAGGGCAGCATCCCCGACAGATTCAGCGCCCAGCAGTTCAGCGACTACCACAGCGAGCTGAACATGAGCAGCCTGGAACTGGGCGACAGCGCCCTGTACTTCTGCGCCTCTAGCTTCCTGGGCGAGAAAACCGAGGCATTCTTTGGGCAGGGCACCAGACTGACCGTGGTGGAAGATCTGCGGAACGTGACCCCCCCCAAGGTGTCCCTGTTCGAGCCTAGCAAGGCCGAGATCGCCAACAAGCAGAAAGCCACACTCGTGTGCCTGGCCAGAGGCTTCTTCCCCGACCACGTGGAACTGTCTTGGTGGGTCAACGGCAAAGAGGTGCACAGCGGCGTGTCCACCGATCCTCAGGCCTACAAAGAGAGCAACTACAGCTACTGCCTGAGCAGCAGACTGCGGGTGTCCGCCACCTTCTGGCACAACCCCCGGAACCACTTCAGATGCCAGGTGCAGTTTCACGGCCTGAGCGAAGAGGACAAGTGGCCCGAGGGCAGCCCTAAGCCCGTGACCCAGAATATCTCTGCCGAGGCCTGGGGCAGAGCCGACTGTGGAATTACCAGCGCCAGCTACCACCAGGGCGTGCTGTCTGCCACCATCCTGTACGAGATCCTGCTGGGCAAGGCCACCCTGTACGCCGTGCTGGTGTCTGGCCTGGTGCTGATGGCCATGGTCAAGAAGAAGAACAGCGGCAGCGGCGCCACCAACTTCAGCCTGCTGAAACAGGCCGGCGACGTGGAAGAGAACCCTGGCCCTATGACCAGCATCCGGGCCGTGTTCATCTTCCTGTGGCTGCAGCTGGACCTCGTGAACGGCGAGAACGTGGAACAGCACCCCAGCACCCTGAGCGTGCAGGAAGGCGATAGCGCCGTGATCAAGTGCACCTACAGCGACTCCGCCAGCAACTACTTCCCCTGGTACAAGCAGGAACTGGGAAAGCGGCCCCAGCTGATCATCGACATCCGGTCCAACGTGGGAGAGAAGAAGGACCAGCGGATCGCCGTGACCCTGAACAAGACCGCCAAGCACTTCTCCCTGCACATCACCGAGACACAGCCCGAGGACTCCGCCGTGTACTTTTGTGCCGCTTCTGCCGGCGGATCCCAGGGCAATCTGATCTTCGGCAAGGGCACCAAGCTGAGCGTGAAGCCCAACATCCAGAACCCCGAGCCTGCCGTGTACCAGCTGAAGGACCCCAGAAGCCAGGACTCCACCCTGTGCCTGTTCACCGACTTCGACAGCCAGATCAACGTGCCCAAGACCATGGAATCCGGCACCTTCATCACCGACAAGACCGTGCTGGACATGAAGGCCATGGACAGCAAGAGCAACGGCGCCATTGCCTGGTCCAACCAGACCAGCTTCACATGCCAGGACATCTTCAAAGAGACAAACGCCACCTACCCCAGCAGCGACGTGCCCTGTGATGCCACACTGACCGAGAAGTCCTTCGAGACAGACATGAACCTGAACTTCCAGAACCTGTCCGTGATGGGCCTGAGAATCCTGCTGCTGAAGGTGGCCGGCTTCAATCTGCTGATGACCCTGCGGCTGTGGTCCTCCTGATGAGAATTCCGCTGGGCCTCATGGGCCTTCCGCTCACTGCCCGCTTTCCAG |
